# Analysis of multidimensional factors in attempts to quit using tobacco by Korean adolescents

**DOI:** 10.1186/s12199-020-00913-1

**Published:** 2020-11-20

**Authors:** Mi-Jung Kang, Hyunjin Lee, Mirae Jo

**Affiliations:** College of Nursing, Department of Nursing, Eulji University, Daejeon, 34824 Republic of Korea

**Keywords:** Quit attempt, Adolescent, Tobacco user, Multidimensional factors

## Abstract

**Background:**

This study aims to understand the extent of adolescents’ attempts to quit using tobacco and the factors influencing such attempts in Korea, using a descriptive, cross-sectional design and secondary data analysis with the 2019 Youth Health Behavior Survey.

**Methods:**

The participants were 4028 adolescent tobacco users who had used tobacco for 1 day or more in the past 30 days. The data analysis was performed using IBM SPSS/WIN 26.0 program, and multivariable logistic regression analysis was conducted using the complex sampling method module.

**Results:**

A total of 68.2% of the participants attempted to quit using tobacco. We analyzed the factors for adolescents’ attempts to quit using tobacco by dividing them into psychological, physical, behavioral, and environmental dimensions. The factors influencing adolescents’ attempts to quit using tobacco, identified through multivariable logistic regression analysis, are as follows: participation in sports activities (OR = 1.20, 95% CI 1.01–1.41), vigorous physical activity (OR = 1.24, 95% CI 1.06–1.46), and type of tobacco product used (OR = 1.65, 95% CI 1.24–2.21) in the behavioral dimension; pictorial cigarette pack warnings (perceived smoking as unhealthy) (OR = 1.91, 95% CI 1.56–2.36), and the presence of secondhand smoking at home (OR = 1.18, 95% CI 1.01–1.38) in the environmental dimension.

**Conclusions:**

Schools and public healthcare providers must consider multidimensional factors when providing support for successful tobacco cessation in adolescents and focus particularly on elements relating to physical activity and environmental factors.

## Background

As it celebrates the 2020 World No Tobacco Day, the World Health Organization (WHO) is engaging in a campaign to “protect children and young people from tobacco and related industries” [1]. Smoking is a risky behavior that blocks the oxygen supply to multiple organs, including the lungs, leading to functional damage [1]. About 90% of smokers begin smoking before the age of 18 [2, 3]. According to the Korea Ministry of Health and Welfare, adolescents in Korea smoke to relieve stress, escape boring situations, and find new stimuli [4]. Once this becomes a habit, they feel the craving for tobacco and continue to smoke [4]. However, as smoking in one’s adolescence continues into adulthood, it may contribute to the development of drug abuse, depression, and other problematic behaviors [5]. Some studies even report that the rate of premature death in smokers is more than six times higher than the average [6].

According to the 15th Youth Health Behavior Survey, conducted in 2019, the rate of tobacco use in Korean adolescents was 9.3% in males and 3.8% in females [7]. Despite various preventative measures, including warnings on the risks of using tobacco, as well as tobacco-cessation education, only 70.1% of the male students who used tobacco attempted to quit in the previous 12 months. Those rates were higher than the 66.5% of female students who attempted to quit smoking [7].

In Korea, the number of adolescents who smoke cigarettes is ten times the number of those who only use e-cigarettes or heated tobacco products (HTPs) [7], with approximately 75% of adolescents using both e-cigarettes and cigarettes [8]. Adolescents who use e-cigarettes have been shown to smoke more frequently than adolescents who smoke regular cigarettes [9]. In some countries, e-cigarettes are reported to be used for smoking cessation [10, 11]; however, according to the WHO, the cessation effect of e-cigarettes has not yet been systematically evaluated [12]. Korean adolescents who use e-cigarettes or HTPs have stated that they are used because they are perceived as less harmful to one’s health than cigarettes, do not smell, and can be smoked freely at school or home [13].

According to a study by Myers et al. [14], 60.9% of the high school students who smoke daily have attempted to quit. However, relapse is common in the process of quitting smoking, which highlights the importance of the factors that influence this process. Furthermore, smoking is a multidimensional behavior relating to genetic, psychological, environmental, and sociocultural characteristics [15]. Therefore, it is necessary to identify the factors influencing smoking and attempts to quit smoking from a multidimensional perspective to reduce rates of adolescent smoking.

Previous studies that have examined smoking and quitting smoking in adolescents have generally focused on factors such as behavior [16–21], perception and attitude [3, 22], psychology [23] and environment [18, 23], and other specific subjects [18, 24–26], indicating a general lack of studies from the multidimensional perspective. In addition, no studies have distinguished physical characteristics, including physical disorders and behavioral characteristics.

Thus, we aimed to identify the factors that influence attempts to quit using tobacco from a multidimensional perspective of psychological, physical, behavioral, and environmental dimensions to reduce rates of using tobacco in Korean adolescents.

## Method

### Research design

This study is descriptive, cross-sectional research involving a secondary analysis of the data from the 15th Youth Health Behavior Survey conducted in 2019 [7].

### Research participants and data

The Youth Health Behavior Survey is an annual, official statistics survey that is conducted online by the Ministry of Education, the Ministry of Health and Welfare, and the Korea Centers for Disease Control and Prevention to understand the status of, and trends in, health behavior among Korean adolescents [7]. The 15th Youth Health Behavior Survey utilized middle and high school data nationwide, as of April 2019. Samples were extracted using the complex sample design method. The sample extraction process resulted in 60,100 students, and a total of 57,303 students participated in the final survey [7]. The participants of this study were classified as adolescent tobacco users if any of them had used one or more of the following for one or more days in the past 30 days: (1) cigarettes, (2) e-cigarettes with nicotine, or (3) any HTPs. In total, 4028 adolescent tobacco users were selected for analysis from the original sample of 57,303 participants.

### Definition of research variables

#### General characteristics

The general characteristics of the subjects consist of gender, school type, economic status, and academic performance.

#### Psychological characteristics

The psychological characteristics of the participants included perceived stress, depressive feelings, suicidal ideation, sleep satisfaction, subjective health status, and body shape perception. Stress perception was classified as “high” and “low.” “Yes” or “No” responses were used to gauge depressive feelings and suicidal ideation. Sleep satisfaction was classified based on responses to the question, “Do you feel that the amount of sleep that you had in the past seven days to be sufficient to recover from fatigue?” Subjective health status classified “Healthy,” “Fair,” and “Unhealthy,” and body shape perception classified the responses to the question, “What do you think about your body shape?”

#### Physical characteristics

Participants’ physical characteristics consisted of having a diagnosis of asthma, allergic rhinitis, or atopic dermatitis, as well as obesity and experience of halitosis. “Yes” or “No” responses were used to respond to questions regarding any past or current (1) diagnosis of asthma, (2) allergic rhinitis, and (3) atopic dermatitis. For obesity, body mass index (BMI) was calculated as weight (kg)/height (m^2^) and in accordance with the guidelines from the Korean Society for the Study of Obesity [27]. The experience of halitosis was based on “Yes” or “No” responses to the question, “Have you experienced any of the symptoms in the past 12 months?”

#### Behavioral characteristics

Participants’ behavioral characteristics included participation in sports activities, vigorous physical activity, weight loss attempts, current drinking, and type of tobacco product used. The participation in sports activities was based on the question, “How many sports teams have you regularly participated in school this semester?” Vigorous physical activity was based on the question, “How many days in the past seven days have you engaged in vigorous physical activity for more than 20 minutes, resulting in being significantly short of breath or sweating?” Weight loss attempts were assessed by the question, “Have you attempted to control your weight in the past 30 days?” Current drinking was measured using the question, “How many days in the past 30 days have you drank one or more drink?” Type of tobacco product used was classified into “only cigarettes” and “e-cigarettes with nicotine or HTPs (without cigarettes),” and those who used both cigarettes and e-cigarettes with nicotine or HTPs were classified as “dual use.”

#### Environmental characteristics

Environmental characteristics consist of pictorial cigarette pack warning-recognition of health hazards and secondhand smoking at home. Pictorial cigarette pack warning-recognition of health hazards classified the responses to the question, “Do you think smoking is bad for you after looking at the warning images on the cigarette packs?” Secondhand smoking at home used the question, “How many days have you breathed smoke from cigarettes someone else smoked inside your home in the past seven days?”

#### Attempts to quit using tobacco

Attempts to quit using tobacco were recorded as “Yes” or “No” responses to the question, “Have you tried to quit using tobacco (i.e., cigarette, e-cigarette with nicotine, HTPs) used in the past 12 months?”

### Data analysis method

The data analysis was conducted using the IBM SPSS/WIN 26.0 program. As the sample from the Youth Health Behavior Survey was extracted under the complex sampling design, calculations were done in consideration of stratification variables, cluster variables, weighting, and finite population correction. The data were analyzed using the complex sampling method module. Descriptive statistics and cross-tabulation analysis were utilized to confirm the distribution of variables in the participants’ general characteristics; psychological, physical, behavioral, and environmental domains; and attempts to quit using tobacco, and calculated unweighted frequency and weighted percentage. Through univariate logistic regression analysis, the relationship between independent variables and attempts to quit using tobacco was determined, and variables were identified to include in multivariable analyses. Multivariable logistic regression analysis was performed to identify factors that influence attempts to quit using tobacco, and 95% confidence interval (CI) and the adjusted odds ratio (AOR) were calculated.

## Results

### Participant characteristics

In terms of general participant characteristics, 73.0% of the participants were male, and 76.4% were high school students. Economic status was described by 44.5% as “middle,” and 50.5% of the participants had “low” academic performance. In terms of the psychological characteristics, 48.5% of the participants responded “High” for perceived stress. In the past 12 months, 42.7% had experienced depressive feelings, and 23.6% considered suicide. Sleep time was reported by 55.7% of the participants as being insufficient to recover from fatigue, and 67.8% responded that they were in good health. Body shape perception was described by 37.3% participants as being “Fat.” In terms of physical characteristics, 10.8% of the subjects were diagnosed with asthma, 35.0% were diagnosed with allergic rhinitis, and 22.3% were diagnosed with atopic dermatitis. With regard to weight, 19.8% of the participants were obese, and 22.9% had experience with halitosis. In terms of behavioral characteristics, 49.8% of the participants reported that they regularly participated in sports teams, and 41.3% engaged in vigorous physical activity for 3 days or more per week. In the past 30 days, 33.2% had attempted to lose weight, and 67.7% of the participants were currently consuming alcohol. In terms of the type of tobacco product used, 46.3% of the participants used “only cigarettes,” 45.2% reported “dual use,” and 8.5% reported using “e-cigarette with nicotine or HTPs” (without cigarettes). In our study, 0.7% (*n* = 25) of participants reported only using e-cigarette with nicotine, and 0.4% (*n* = 15) were HTPs-only users. Most of the e-cigarette users also reported using HTPs. In terms of environmental characteristics, 85.0% of the participants perceived smoking to be unhealthy by seeing the pictorial warnings on the cigarette packs, and 43.6% experienced secondhand smoking at home in the past 7 days (Table 1).

**Table 1:** Sample characteristics (*N* = 4028)

Categories	Variables	Subcategories	Total	
			***n***^**b**^	%^**c**^
General characteristics	Gender	Male	2889	73.0
		Female	1139	27.0
	School type	Middle school	1034	23.6
		High school	2994	76.4
	Economic status	High	1460	36.5
		Middle	1752	44.5
		Low	816	19.0
	Academic performance	High	1034	25.5
		Middle	968	24.0
		Low	2026	50.5
Psychological dimensions	Perceived stress	High	1955	48.5
		Low	2073	51.5
	Depressive feelings	Yes	1751	42.7
		No	2277	57.3
	Suicidal ideation	Yes	976	23.6
		No	3052	76.4
	Sleep satisfaction	Enough	610	15.0
		Fair	1192	29.3
		Not enough	2226	55.7
	Subjective health status	Healthy	2715	67.8
		Fair	906	22.2
		Unhealthy	407	10.0
	Body shape perception	Skinny	1109	28.0
		Normal	1396	34.7
		Fat	1523	37.3
Physical dimensions	Asthma	Yes	426	10.8
		No	3602	89.2
	Allergic rhinitis	Yes	1416	35.0
		No	2612	65.0
	Atopic dermatitis	Yes	905	22.3
		No	3123	77.7
	Obesity	Yes	743	19.8
		No	3015	80.2
	Experiences of halitosis	Yes	944	22.9
		No	3084	77.1
Behavioral dimensions	Participation in sports activities	Yes	2050	49.8
		No	1978	50.2
	Vigorous physical activity (≥ 3 days/week)	Yes	1664	41.3
		No	2364	58.7
	Weight loss attempt	Yes	1345	33.2
		No	2683	66.8
	Current drinking	Yes	2719	67.7
		No	1309	32.3
	Type of tobacco product used	Only cigarettes	1903	46.3
		E-cigarettes with nicotine or HTPs (without cigarettes)	335	8.5
		(Only e-cigarettes with nicotine)	25	0.7
		(Only HTPs)	15	0.4
		Dual use	1790	45.2
Environmental dimensions	Pictorial cigarette pack warnings recognized for health hazards^a^	Yes	3071	85.0
		No	526	15.0
	Secondhand smoking at home (≥1 day/week)	Yes	1778	43.6
		No	2250	56.4

*HTPs* heated tobacco products, *Dual use* cigarettes and e-cigarettes with nicotine or HTPs

^a^Missing data were not included

^b^Unweighted number

^**c**^Weighted percent

### Differences in rates of attempts to quit using tobacco by related factors in adolescent tobacco users

The rate of attempts to quit using tobacco across all participants was 68.2%. The results of the cross-tabulation analysis and the univariate logistic regression are shown in Table 2. A univariate logistic regression analysis was used to determine the association of each factor with the attempts to quit using tobacco. As a result of the analysis, “high” academic performance (OR = 0.75; 95% CI 0.0.64–0.89), low stress perception (OR = 1.19; 95% CI 1.03–1.38), asthma (OR = 0.77; 95% CI 0.63–0.94), obesity (OR = 1.27; 95% CI 1.07–1.50), participating in sports activities (OR = 1.24; 95% CI 1.08–1.42), practicing vigorous physical activity (OR = 1.25; 95% CI 1.10–1.43), only cigarette use (OR = 1.62; 95% CI 1.29–2.04), dual use of cigarettes and e-cigarettes or HTPs (OR = 1.59; 95% CI 1.26–2.00), recognizing smoking is unhealthy from the warnings on the cigarette packs (OR = 1.93; 95% CI 1.60–2.34), and no experience of secondhand smoke at home (OR = 1.25; 95% CI 1.10–1.44) were significantly associated with attempts to quit using tobacco.

**Table 2:** Differences in attempts to quit using tobacco by related factors in adolescent tobacco users

Categories	Variables	Subcategories	Attempts to quit using tobacco		Univariate logistic regression	
			Yes			
			***n***^**b**^	***%***^***c***^	OR (95% CI)	***p***
General characteristics	Total		2749	68.2		
	Gender	Male	2003	69.1	1.16 (0.99–1.36)	.054
		Female	746	65.8	Reference	
	School type	Middle school	718	70.0	1.12 (0.95–1.32)	.179
		High school	2031	67.6	Reference	
	Economic status	High	975	66.6	0.96 (0.81–1.15)	.676
		Middle	1226	69.8	1.11 (0.93–1.32)	.231
		Low	548	67.5	Reference	
	Academic performance	High	660	64.1	0.75 (0.64–0.89)	.001
		Middle	672	68.0	0.90 (0.74–1.08)	.247
		Low	1417	70.4	Reference	
Psychological dimensions	Perceived stress	High	1294	66.2	Reference	
		Low	1455	70.1	1.19 (1.03–1.38)	.018
	Depressive feelings	Yes	1194	68.4	Reference	
		No	1555	68.0	0.98 (0.86–1.12)	.775
	Suicidal ideation	Yes	639	66.1	Reference	
		No	2110	68.8	1.13 (0.95–1.35)	.169
	Sleep satisfaction	Enough	404	68.1	1.05(0.84–1.30)	.688
		Fair	845	70.4	1.17 (0.99–1.37)	.055
		Not enough	1500	67.1	Reference	
	Subjective health status	Healthy	1876	68.8	1.22 (0.98–1.52)	.073
		Fair	616	68.0	1.18 (0.91–1.52)	.213
		Unhealthy	257	64.4	Reference	
	Body shape perception^a^	Skinny	767	68.9	1.12 (0.96–1.31)	.164
		Normal	964	69.5	1.15 (0.99–1.34)	.069
		Fat	1018	66.5	Reference	
Physical dimensions	Asthma	Yes	270	62.9	0.77 (0.63–0.94)	.009
		No	2479	68.8	Reference	
	Allergic rhinitis	Yes	957	66.7	0.90 (0.77–1.04)	.960
		No	1792	69.0	Reference	
	Atopic dermatitis	Yes	601	66.6	0.91 (0.77–1.07)	.246
		No	2148	68.7	Reference	
	Obesity	Yes	486	64.9	Reference	
		No	2109	70.1	1.27 (1.07–1.50)	.007
	Experiences of halitosis	Yes	647	69.2	1.06 (0.91–1.24)	.454
		No	2102	67.9	Reference	
Behavioral dimensions	Participation in sports activities	Yes	1441	70.5	1.24 (1.08–1.42)	.002
		No	1308	65.9	Reference	
	Vigorous physical activity (≥ 3 days/week)	Yes	1178	71.0	1.25 (1.10–1.43)	.001
		No	1571	66.2	Reference	
	Weight loss attempt	Yes	932	69.4	1.09 (0.94–1.27)	.257
		No	1817	67.6	Reference	
	Current drinking	Yes	1854	67.8	Reference	
		No	895	69.1	1.06 (0.91–1.24)	.440
	Type of tobacco product used	Only cigarettes	1323	69.3	1.62 (1.29–2.04)	< .001
		E-cigarettes with nicotine or HTPs (without cigarettes)	191	58.3	Reference	
		Dual use	1235	68.9	1.59 (1.26–2.00)	< .001
Environmental dimensions	Pictorial cigarette pack warnings (perceived smoking as unhealthy)^a^	Yes	2229	72.9	1.93 (1.60–2.34)	< .001
		No	307	58.1	Reference	
	Secondhand smoking at home (≥ 1 day/week)^a^	Yes	1163	65.4	Reference	
		No	1586	70.4	1.25 (1.10–1.44)	.001

*Dual use* cigarettes and e-cigarettes with nicotine or HTPs, *OR* odds ratio, *CI* confidence interval, *HTPs* heated tobacco products

^a^Missing data were not included

^b^Unweighted number

^c^Weighted percent

### Factors influencing attempts to quit using tobacco in adolescent tobacco users

Multivariable logistic regression analysis was performed by entering significant association variables in univariate analysis (Table 3). As a result of the analysis, participating in sports activities (AOR = 1.20; 95% CI 1.01–1.41), practicing vigorous physical activity (AOR = 1.24; 95% CI 1.06–1.46), only cigarette use (AOR = 1.65; 95% CI 1.24–2.21), the dual use of cigarettes and e-cigarettes or HTPs (AOR = 1.62; 95% CI 1.22–2.15), recognizing smoking is unhealthy from the pictorial warnings on the cigarette packs (AOR = 1.91; 95% CI 1.56–2.36), and no experience of secondhand smoke at home (AOR = 1.18; 95% CI 1.01–1.38) were significantly associated with attempts to quit using tobacco.

**Table 3:** Factors influencing attempts to quit using tobacco in adolescent tobacco users

Categories	Variables	Subcategories	AOR (95% CI)^**a**^	***p***
General characteristics	Academic performance	High	0.87 (0.72–1.06)	.171
		Middle	0.85 (0.69–1.04)	.112
		Low	Reference	
Psychological dimension	Perceived stress	High	Reference	
		Low	1.12 (0.94–1.32)	.197
Physical dimensions	Asthma	Yes	1.25 (0.98–1.59)	.076
		No	Reference	
	Obesity	Yes	Reference	.164
		No	1.15 (0.95–1.39)	
Behavioral dimensions	Participation in sports activities	Yes	1.20 (1.01–1.41)	.032
		No	Reference	
	Vigorous physical activity (≥ 3 days/week)	Yes	1.24 (1.06–1.46)	.009
		No	Reference	
	Type of tobacco product used	Only cigarettes	1.65 (1.24–2.21)	.001
		E-cigarettes with nicotine or HTPs (without cigarettes)	Reference	
		Dual use	1.62 (1.22–2.15)	.001
Environmental dimensions	Pictorial cigarette pack warnings (perceived smoking as unhealthy)	Yes	1.91 (1.56–2.36)	< .001
		No	Reference	
	Secondhand smoking at home (≥ 1 day/week)	Yes	Reference	.035
		No	1.18 (1.01–1.38)	

*CI* confidence interval, *AOR* adjusted odds ratio

^a^Nagelkerke *R*^2^ = .035, Cox and Snell *R*^2^ = .024; adjusted for academic performance, perceived stress, asthma, obesity, participation in sports activities, vigorous physical activity (≥ 3 days/week), type of tobacco product used, pictorial cigarette pack warnings being used to recognize cigarettes as unhealthy, secondhand smoking at home (≥ 1 days/week)

## Discussion

Adolescent tobacco users are at a significant risk of becoming subsequent tobacco users [28]. In addition, tobacco may be a gateway to substance abuse, depression, and other problematic behaviors [5, 29]. Therefore, the control of tobacco is especially important for adolescents.

This study has confirmed factors across multiple dimensions relating to attempts to quit using tobacco in Korean adolescents. The study found that the rate of attempts to quit in adolescent tobacco users was 68.2%. These results represent a 3.3% drop from 71.3% in 2018 [7]. A recent study of 3.3 million American adolescents also indicated that only 57.5% of adolescent tobacco users had attempted to quit using tobacco [30].

In the past 10 years, the rate of adolescent tobacco use in Korea dropped significantly, from 12.1% in 2010 to 6.7% in 2019 [31]. This decline corresponds to efforts in preventing adolescent tobacco use, which have taken place since 2010. Several tobacco-cessation policies and stronger regulations on tobacco use were put in place, such as strengthening tobacco-cessation education in schools, expanding no-tobacco-use zones, raising tobacco prices, and placing warning images on cigarette packs [32]. While the rate of adolescent tobacco use has decreased, the rate of quit attempts in adolescent tobacco users appear to be similar to, or lower than, the rates seen in existing studies [9]. This may be related to levels of nicotine dependence throughout the period of tobacco use [33].

This study has classified the factors influencing tobacco-use quit attempts in adolescent tobacco users into physical, psychological, behavioral, and environmental dimensions for analysis and found that behavioral and environmental dimensions effected significant results. Specifically, the influencing factors of behavioral dimensions were sports team participation, vigorous physical activity, and the type of tobacco product used; the factors in the environmental domain were warning images on cigarette packs and experience of secondhand smoking at home.

Adolescent tobacco users who participated in sports teams and engaged in vigorous physical exercise were more likely to attempt to quit using tobacco compared to their peers who did not. This is in line with the results of existing studies that indicated physical activity is related to tobacco-use quit attempts [9]. Existing studies have found that levels of stress and depression were higher in adolescent tobacco users compared to non-tobacco users. This was generally caused by the burden of academics and the lack of coping abilities required to deal with stress [34]. Particularly, Korean adolescents are choosing tobacco use as a method to relieve stress [4], as well as to associate with their peer groups [13]. Therefore, appropriate interventions are needed at the school and at the regional community level to relieve the stress faced by adolescents. Physical activity in adolescents has a positive influence on their mental health [35]. Therefore, in addition to strengthening tobacco-cessation education, it is necessary to provide adolescents the opportunities to form groups and participate in various sports activities or other areas of interest.

In this study, the adolescents who used e-cigarettes or HTPs (without cigarettes) had lower rates of tobacco-use quit attempts compared to those using cigarettes. According to recent reports, e-cigarettes or HTP users are often used as smoking aids because of the perception that e-cigarettes or HTPs are not as harmful to health as cigarettes, do not smell, and are convenient for smoking freely at school and home [13, 36]. Adolescent tobacco users may try e-cigarettes or HTPs as a tobacco-use cessation aid; however, they fail to quit and end up becoming dual users [13]. Similarly, the number of adolescent dual users have increased overseas [37], underscoring the need to review the use of e-cigarettes and HTPs. Recent research has suggested the negative health effects of e-cigarettes [36, 38], indicating the need to clearly understand the conflicting evaluations on the effects of e-cigarettes and HTPs. In addition, the diversity of e-cigarette and HTPs is making consensus or regulation of these products difficult. In the future, it will be necessary to specifically examine administrative regulations on the production and sale of e-cigarettes and HTPs based on medical evidence. These efforts may help prevent and reduce tobacco use by adolescents.

The recognition of health hazards via warning images on cigarette packs appears to be the most significant factor in tobacco-use quit attempts in adolescent tobacco users. These have been found to have a positive influence helping adolescents understand the risks of using tobacco [39]. Additionally, viewing health warning signs typically induces health behavior; thus, emphasizing these warning signs as much as possible is of utmost importance [40]. The warning images on cigarette packs have also been found to be more effective for adolescent non-tobacco users compared to tobacco users [32] and may be effective in encouraging tobacco-use prevention in adolescents. As the age of tobacco users continues to decrease in adolescents, it is also necessary to develop more effective warning images and messages that target this age group.

In this study, adolescents who experienced secondhand smoke at home had fewer tobacco-use quit attempts compared to those who had not experienced secondhand smoke at home. Adolescents are more likely to smoke if a member of their family smokes and are more likely to adopt an accepting attitude toward smoking [41]. Exposure to secondhand smoking at home has been found to influence asthma, depression, and perceived stress in adolescents, which should act as a warning against smoking at home by adult smokers [42]. Furthermore, exposure to secondhand smoking at home is associated with female and younger adolescents, as they tend to spend more time at home compared to older, male adolescents, making them more exposed to secondhand smoke when there is a smoker in their family [9]. Therefore, family members quitting smoking is necessary to prevent adolescents from using tobacco and for adolescent tobacco users to try to quit using tobacco.

Our research has several limitations. First, there was no information on the individual duration of tobacco-use quit attempts, as this was not included in the raw datasets. Therefore, we were unable to evaluate the relationship between factors influencing tobacco-use quit attempts in adolescent tobacco users and the periods of successful tobacco cessation. Second, cigarette smokers, e-cigarette users, and HTP users were classified into one tobacco user group. Some people can use an e-cigarette to quit smoking. However, according to the results of previous studies in Korea, adolescents who use e-cigarettes showed a pattern of smoking more frequently and using more cigarettes [9]. In addition, when HTPs were used, the purpose of the use was not smoking; there were many cases where it was used as a substitute for smoking [36]. In our study, the number of exclusive e-cigarette users was considerably small, and most of the e-cigarette users were also using HTPs. Finally, it was not possible to clearly determine whether the e-cigarette users may be a group that previously used tobacco. It is necessary to investigate the purpose of e-cigarette use when collecting data from The Youth Health Behavior Surveys conducted in the future.

Despite these potential limitations, this study utilized national statistical data that represent the health behaviors of Korean adolescents to explore various aspects of the factors influencing tobacco-use quit attempts. As highlighted in existing literature, adolescent tobacco users need to be continuously monitored and provided with appropriate interventions.

However, most tobacco-cessation policies for adolescents that are currently implemented by the Korean government are mainly regulatory policies [43]. This study confirmed that, although regulatory and deterrence policies related to the environmental dimension were showing an effect, adolescents’ healthy behavior, such as participation in sports activities and vigorous physical activities, was also an important factor influencing attempts to quit using tobacco. Therefore, it is urgent to develop specific policies encouraging individual healthy behaviors to promote Korean adolescent tobacco users’ attempts to quit tobacco.

As adolescents have been using tobacco for shorter periods compared to adult tobacco users, they have lower levels of nicotine dependence [33], which may increase the likelihood of successful cessation of tobacco use. Schools and communities must provide various physical activity programs in addition to tobacco-cessation education, which can help develop an environment where adolescent tobacco users can escape the temptation of using tobacco. As government support and policy can influence tobacco-use cessation in adolescents, it is necessary to develop clear tobacco-cessation policies (i.e., clear agreement and regulation over the diverse range of e-cigarette products) and content that can induce attempts to quit among adolescent tobacco users.

## Conclusion

This study identified factors relating to attempts to quit using tobacco in Korean adolescent tobacco users in multiple dimensions. We analyzed the data of 4028 adolescent tobacco users found in the 2019 Korea Youth Health Behavior Survey in terms of physical, psychological, behavioral, and environmental dimensions. We confirmed that Korean adolescent tobacco users’ attempts to quit using tobacco are related to behavioral and environmental dimensions. This study is meaningful in that it has provided evidence-based results for the practical application of tobacco-cessation policy for adolescents in Korea.

## Data Availability

Please contact the corresponding author for data requests.

## Abbreviations

BMI: Body mass index
OR: Odds ratio
CI: Confidence interval
AOR: Adjusted odds ratio
HTPs: Heated tobacco products

## References

[1] World Health Organization (2020). Stop tobacco industry exploitation of children and young people.

[2] Trofor A, Mihaicuta S, Man MA, Miron R, Esanu V, Trofor L (2010). Approaching tobacco dependence in youngsters: impact of an interactive smoking cessation program in a population of Romanian adolescents. J Clin Exp Investig.

[3] Amrock SM, Weitzman M (2015). Adolescents’ perceptions of light and intermittent smoking in the United States. Pediatrics.

[4] Korea Ministry of Health and Welfare (2017). No smoke guide.

[5] Kendler KS, Myers J, Damaj MI, Chen X (2013). Early smoking onset and risk for subsequent nicotine dependence: a monozygotic co-twin control study. Am J Psychiatry.

[6] Lee EH, Park SK, Ko KP, Cho IS, Chang SH, Shin HR, Kang D, Yoo KY (2013). Cigarette smoking and mortality in the Korean Multi-center Cancer Cohort (KMCC) Study. J Prev Med Public Health.

[7] Korea Ministry of Education. Korea Ministry of Health and Welfare, Korea Centers for Disease Control and Prevention. The statistics report of the Fifteenth Korea Youth Risk Behavior Web-based Survey. Cheongju, South Korea: Korea Centers for Disease Control and Prevention. 2019. http://www.cdc.go.kr/yhs/home.jsp. Accessed 8 June 2020.

[8] Lee S, Grana RA, Glantz SA (2014). Electronic cigarette use among Korean adolescents: a cross-sectional study of market penetration, dual use, and relationship to quit attempts and former smoking. J Adolesc Health.

[9] Park S, Lee H, Min S (2017). Factors associated with electronic cigarette use among current cigarette-smoking adolescents in the Republic of Korea. Addict Behav.

[10] Hartmann-Boyce J, McRobbie H, Bullen C, Begh R, Stead LF, Hajek P (2016). Electronic cigarettes for smoking cessation. Cochrane Database Syst Rev.

[11] Brown J, Beard E, Kotz D, Michie S, West R (2014). Real-world effectiveness of e-cigarettes when used to aid smoking cessation: a cross-sectional population study. Addiction..

[12] World Health Organization Framework Convention on Tobacco Control. Electronic nicotine delivery systems. Geneva: World Health Organization Framework Convention on Tobacco Control. 2014. https://www.who.int/tobacco/framework/WHO_FCTC_english.pdf?ua=1. Accessed 23 Aug 2020.

[13] Lee YR, Kim H, Lee S, Jeon J, Chu S, Jee SH (2017). Associations between the attempts for quitting smoking and electronic cigarette use in Korean adolescent. Korean J Health Educ Promot.

[14] Myers MG, Gwaltney CJ, Strong DR, Ramsey SE, Brown RA, Monti PM, Colby SM (2017). Adolescent first lapse following smoking cessation: Situation characteristics, precipitants and proximal influences. Addict Behav.

[15] Wang M, Zhong JM, Fang L, Wang H (2016). Prevalence and associated factors of smoking in middle and high school students: a school-based cross-sectional study in Zhejiang Province, China. BMJ Open.

[16] Ra JS, Kim HS, Cho YH (2018). Factors associated with intermittent and light smoking among Korean high school students: intermittent and light smoking among Korean adolescents. J Korean Acad Community Health Nurs.

[17] Sohn BD (2015). The roles of structural components giving adolescents helpful information about quitting smoking. Korean J Youth Stud.

[18] Lee Y (2016). Daily smoking girls’ tobacco use, health behaviors and family factors: analysis of 2015 Korean youth risk behavior web-based survey. J Digital Convergence.

[19] Yim SY, Park MH (2017). Comparison of the factors affecting smoking quit attempts in adolescent smokers according to amount of smoking. J Korea Contents Assoc.

[20] Kim SR (2016). Influencing factors on smoking quit attempts in adolescent smokers: comparison between daily smokers and non-daily smokers [Doctoral Dissertation].

[21] Lee BR (2016). Investigation of smoking behavior and quitting attempts in Korean adolescent smokers guided by the problem behavior theory [master’s thesis].

[22] De Houwer J, Custers R, De Clercq A (2006). Do smokers have a negative implicit attitude toward smoking?. Cognit Emot.

[23] Park J (2016). Factors affecting attempts to quit smoking in Korean adolescents. J Korean Soc Sch Health.

[24] Seo YS, Kim YI (2013). Factors affecting smoking middle school students’ intention to quit smoking: on the basis of the ASE model. J Korean Acad Community Health Nurs.

[25] Park S, Suh G, Park S, Chung J (2018). Factors affecting attempts to quit smoking in smoking high school students. J Korea Inst Youth Facil Environ.

[26] Her W (2019). Exploring Korean American young adults’ smoking behavior regarding social influences and smoking attitude [doctoral dissertation].

[27] Seo MH, Lee WY, Kim SS, Kang JH, Kang JHM, Kim KK (2019). 2018 Korean Society for the Study of Obesity guideline for the management of obesity in Korea. J Obes Metab Syndr.

[28] Mathers M, Toumbourou JW, Catalano RF, Williams J, Patton GC (2006). Consequences of youth tobacco use: a review of prospective behavioural studies. Addiction.

[29] World Health Organization. Health effects of smoking among young people. https://www.who.int/tobacco/control/populations/youth_health_effects/en/. Accessed 8 July 2020.

[30] Wang TW, Gentzke AS, Creamer MR, Cullen KA, Holder HE, Sawdey MD (2019). Tobacco product use and associated factors among middle and high school students—United States, 2019. MMWR Surveill Summ.

[31] Korea Ministry of Education. Korea Ministry of Health and Welfare, Korea Centers for Disease Control and Prevention. The statistics report of the Fifteenth Korea Youth Risk Behavior Web-based Survey, adolescent smoking rate. Daejeon: Korean Statistical Information Service; 2019. http://kosis.kr/statHtml/statHtml.do?orgId=117&tblId=DT_117_12_Y003 &conn_path=I2. Accessed Jun 8 2020.

[32] Hwang JE, Cho SI (2020). The association between new graphic health warning labels on tobacco products and attitudes toward smoking among south Korean adolescents: a national cross-sectional study. BMC Public Health.

[33] Horn K, Fernandes A, Dino G, Massey CJ, Kalsekar I (2003). Adolescent nicotine dependence and smoking cessation outcomes. Addict Behav.

[34] Kim HO, Jeon MS (2007). The relationship between smoking, drinking and the mental health in adolescents. J Korean Public Health Nurs.

[35] Biddle SJ, Ciaccioni S, Thomas G, Vergeer I (2019). Physical activity and mental health in children and adolescents: an updated review of reviews and an analysis of causality. Psychol Sport Exerc.

[36] Lee CM, Kim SR, Cheong YS (2018). Issues of new types of tobacco (e-cigarette and heat-not-burn tobacco): from the perspective of ‘tobacco harm reduction’. J Korean Med Assoc.

[37] Cullen KA, Ambrose BK, Gentzke AS, Apelberg BJ, Jamal A, King BA (2018). Notes from the field: use of electronic cigarettes and any tobacco product among middle and high school students—United States, 2011–2018. MMWR Morb Mortal Wkly Rep.

[38] Cho HJ (2020). Comparison of the risks of combustible cigarettes, e-cigarettes, and heated tobacco products. J Korean Med Assoc.

[39] Hammond D, Reid JL, Driezen P, Boudreau C (2012). Pictorial health warnings on cigarette packs in the United States: an experimental evaluation of the proposed FDA warnings. Nicotine Tob Res.

[40] Hammond D, Fong GT, McNeill A, Borland R, Cummings KM (2006). Effectiveness of cigarette warning labels in informing smokers about the risks of smoking: findings from the International Tobacco Control (ITC) Four Country Survey. Tob Control.

[41] Thrul J, Bühler A, Ferguson SG (2014). Situational and mood factors associated with smoking in young adult light and heavy smokers. Drug Alcohol Rev.

[42] Park S (2017). Associations between household secondhand smoke exposure and health problems among non-smoking adolescents in the Republic of Korea. J Prim Prev.

[43] Korea health Promotion Institute. Tobacco prevention in adolescents. Available online: https://www.khealth.or.kr/board?menuId=MENU00882&siteId=null. Accessed 8 June 2020.

